# The Versatile Roles of Cancer-Associated Fibroblasts in Colorectal Cancer and Therapeutic Implications

**DOI:** 10.3389/fcell.2021.733270

**Published:** 2021-10-01

**Authors:** Longfei Deng, Nianfen Jiang, Jun Zeng, Yi Wang, Hongjuan Cui

**Affiliations:** ^1^Cancer Center, Medical Research Institute, Southwest University, Chongqing, China; ^2^Health Management Center, Southwest University Hospital, Chongqing, China; ^3^Department of Genetics and Cell Biology, College of Life Sciences, Chongqing Normal University, Chongqing, China; ^4^Department of General Surgery, The Ninth People’s Hospital of Chongqing, Affiliated Hospital of Southwest University, Chongqing, China; ^5^State Key Laboratory of Silkworm Genome Biology, Southwest University, Chongqing, China

**Keywords:** cancer-associated fibroblast, colorectal cancer, hallmark, tumor microenvironment, therapeutics

## Abstract

The tumor microenvironment (TME) is populated by abundant cancer-associated fibroblasts (CAFs) that radically influence the disease progression across many cancers, including the colorectal cancer (CRC). In theory, targeting CAFs holds great potential in optimizing CRC treatment. However, attempts to translate the therapeutic benefit of CAFs into clinic practice face many obstacles, largely due to our limited understanding of the heterogeneity in their origins, functions, and mechanisms. In recent years, accumulating evidence has uncovered some cellular precursors and molecular markers of CAFs and also revealed their versatility in impacting various hallmarks of CRC, together helping us to better define the population of CAFs and also paving the way toward their future therapeutic targeting for CRC treatment. In this review, we outline the emerging concept of CAFs in CRC, with an emphasis on their origins, biomarkers, prognostic significance, as well as their functional roles and underlying mechanisms in CRC biology. At last, we discuss the prospect of harnessing CAFs as promising therapeutic targets for the treatment of patients with CRC.

## Introduction

Colorectal cancer (CRC), a term referring to colonic cancer and rectal cancer synonymously, ranks the third most common malignant disease across the world and accounts for 9.2% cancer-related mortality (Bray et al., 2018). Despite achievements made in the innovative medicines and therapeutic methods, the success of effective treatment in CRC patients is hindered to some extent by only targeting tumor cells and ignoring the tumor microenvironment (TME) as an accomplice in nursing disease progression. Indeed, the TME significantly blunts the therapeutic responses, and thus, multitargeting tumor cells and co-opted cells simultaneously in the TME compartments is thought to improve the efficacy of current therapeutics (Wu and Dai, 2017). As the predominant architects of the TME, cancer-associated fibroblasts (CAFs) play a tremendous role in cancer progression, including CRC (Sahai et al., 2020). In recent few years, increasing studies have yielded a mass of updated insights into the biology of CAFs which constitute the CRC. In this review, we aimed to summarize these advancements in this field, mainly including the identification of cellular precursors and molecular markers of CAFs, and verification of their prognostic significance in CRC patients, as well as numerous new discoveries in their versatile roles in key hallmarks of CRC pathogenesis and related novel mechanisms. According to these latest findings, we also analyzed the therapeutic potential and prospect of targeting CAFs in future CRC treatment.

## Cells of Origin of Cancer-Associated Fibroblasts in Colorectal Cancer

It is now becoming increasingly clear that CAFs can originate from diverse potential cellular precursors through distinct mechanisms. As known, normal resident tissue fibroblasts upregulate the expression of smooth muscle α-actin (α-SMA), the most common marker of myofibroblasts, and acquire a myofibroblast-like phenotype upon de novo activation by numerous soluble factors, such as the transforming growth factor-β (TGF-β) and platelet-derived growth factor (PDGF) secreted from the neighboring tumor cells (Vonlaufen et al., 2008; Yin et al., 2013). While local fibroblasts are commonly deemed as the dominating origins of CAFs, additional sources also contribute to the pool of tumor stromal CAFs depending on tumor histological types. Among them, the best-studied CAF precursors are mesenchymal stem cells (MSCs), which are recruited from the adult human tissues including bone marrow and connective tissues, and constitute a large portion of CAFs in some cancers such as breast (Weber et al., 2015), prostate (Jung et al., 2013), gastric (Zhu et al., 2014), and pancreatic cancers (Kabashima-Niibe et al., 2013). In addition, circulating fibrocytes recruited from the bone marrow can migrate into the TME and also give origin to CAFs, as observed in the tumor stroma of breast cancer (Barth et al., 2002) and gastric cancer (Terai et al., 2015). Moreover, epithelial cells adjacent to cancer cells are able to differentiate into CAFs by undergoing epithelial-to-mesenchymal transition (EMT) (Iwano et al., 2002). Similar to this scenario, endothelial cells (ECs) represent other progenitors of CAFs by means of endothelial-to-mesenchymal transition (EndMT) (Zeisberg et al., 2007). The remaining CAF sources, though maybe less common, include adipocytes, pericytes, and smooth muscle cells (SMCs) that possess the capacity to convert into CAFs by transdifferentiation (Chen and Song, 2019). Collectively, these categories of cellular precursors diversify CAF population with overt original heterogeneity.

Cancer-associated fibroblasts are present in high abundance in CRC (Adegboyega et al., 2002; Powell et al., 2005). Although the precise origins of CAFs in CRC have not yet been elucidated explicitly, mounting evidence has suggested that fibroblasts remain the major sources (Table 1). TGF-β is a classic stimulus inducing the differentiation of quiescent fibroblasts into CAFs in the TME. It has been reported that upon induction by CRC cell-derived soluble factors, the TGF-β signaling is activated in CAFs, accompanied by increased expression of TGF-β itself, suggesting a cumulative production of TGF-β within the TME that promotes the transdifferentiation of resident fibroblasts into CAFs (Hawinkels et al., 2014). TGF-β is secreted in a form of latent complex. One study has shown that CRC cell-secreted latent TGF-β could be activated by integrin αvβ6, which is expressed on CRC cells, and subsequently activates fibroblasts to exhibit CAF phenotypes. The integrin αvβ6 appears indispensable for this process, since fibroblast activation is disrupted in the absence of integrin αvβ6 (Peng et al., 2018). These studies indicate that interacting with either tumor cells or secreted soluble factors enables TGF-β activation and favors the generation of CAFs in CRC.

**TABLE 1:** The cellular origins of CAFs in CRC.

Type	Location	Differentiation mechanism	References
Fibroblasts	Local tissue	Stimuli: TGF-β, Nodal, IL-34; Regulators: αvβ6, Snail, TIMP-1, dickkopf-3, PKCζ	Gong et al., 2013; Hawinkels et al., 2014; Li et al., 2018, 2019a; Peng et al., 2018; Ferrari et al., 2019; Franze et al., 2020; Kasashima et al., 2021
MSCs	Bone marrow	Cell-cell contacts mediated by Notch-Jagged1 signaling	Peng et al., 2014
ECs	Endothelium	Tubulin-β3 activation and EndMT	Wawro et al., 2018
HSCs	Perisinusoidal	CXCR4/TGF-β1 axis activation	Tan et al., 2020
MCs	Mesothelium	MMT	Gordillo et al., 2020

*MSCs, mesenchymal stem cells; ECs, endothelial cells; EndMT, endothelial-to-mesenchymal transition; HSCs, hepatic stellate cells; MCs, mesothelial cells; MMT, mesothelial-to-mesenchymal transition.*

Moreover, like TGF-β, another TGF superfamily member Nodal has recently been shown correlated positively with α-SMA expression in human CRC tissues. Through activating TGF-β/Smad/Snail pathway, tumor cell-derived Nodal facilitates the transition of normal fibroblasts into CAFs that function to support the tumor growth of CRC cells *in vitro* and *in vivo* (Li et al., 2019a). Some lines of evidence also show that Snail-positive fibroblasts display CAFs properties (Li et al., 2018), further supporting that Snail is an important regulator of CAF formation derived from fibroblasts. Snail is a TGF-β target gene that mediates some pro-tumorigenic roles of TGF-β signaling (David et al., 2016; Moon et al., 2017), and is also necessary for mediating the pro-tumorigenic effects of fibroblasts on CRC cells (Herrera et al., 2014). It is therefore reasonable to speculate that Nodal-mediated CAF formation via Snail signaling could promote aggressive phenotypes in CRC. Moreover, except Nodal, the interleukin (IL)-34, a cytokine overexpressed by CRC cells, can also stimulate normal fibroblasts to display a cellular phenotype resembling that of CAFs (Franze et al., 2020). Thus, the crosstalk between CRC and fibroblasts mediated by soluble factors, such as Nodal and IL-34, plays a significant role in enhancing CAF formation in the TME of CRC. Probably, other CRC cell-secreted factors may also participate in regulating the differentiation of fibroblasts into CAFs, which warrants further explorations.

Some up-to-date studies have also shown the pivotal roles of cancer stroma in the development of CAFs in CRC. For instance, the increased stromal expression of the tissue inhibitor matrix metalloproteinase-1 (TIMP-1) stimulates the accumulation of CAFs within CRC tissues partly through transdifferentiation of resident fibroblasts (Gong et al., 2013). Additionally, dickkopf-3 expressed in the stroma orchestrates a concomitant activation of Wnt signaling and YAP/TAZ signaling which are coordinated to generate CAFs in CRC (Ferrari et al., 2019). Moreover, stromal loss of protein kinase Cζ (PKCζ) promotes generation of a pro-tumorigenic CAF population in human CRC through a SOX2-dependent mechanism (Kasashima et al., 2021). Hence, cues for converting fibroblasts into CAFs in the TME could stem from both CRC cells and the stroma.

In addition to fibroblasts, recent studies have shown that CAFs in CRC also originate from other sources including MSCs, ECs, pericytes, and mesothelial cells (MCs). It is known that bone marrow-derived MSCs can travel to tumor stroma, where they differentiate into CAFs. In an in vitro co-culture model, CRC cells have been reported to induce differentiation of MSCs into CAFs by cell–cell contacts, which is mediated by Notch-Jagged1 signaling and downstream activation of TGF-β/Smad pathway (Peng et al., 2014). This study provides a molecular mechanism explaining the bone marrow-derived MSCs as sources of CAFs in CRC. Further, ECs undergo conversion into CAFs via the process of EndMT, which is associated with microtubule cytoskeleton reorganization. One study has shown a mechanistic perspective that invasive CRC cells induce the EndMT of ECs to generate CAFs via upregulation and phosphorylation of tubulin-β3, which is mainly dependent on TGF-β stimulation (Wawro et al., 2018). However, whether CRC cells induce transform of ECs *in vivo* needs more investigations. Analogous to activation process following liver damage, the quiescent hepatic stellate cells (HSCs), a subset of liver-specific pericytes, are activated and differentiated into myofibroblasts when tumor micrometastases are developed in liver lobules (Vidal-Vanaclocha, 2008). A recent discovery has represented data showing that CRC cells are able to interact with HSCs and promote SDF-1 secretion, which in turn binds to CXCR4 and induces TGF-β1 expression and secretion in CRC cells, eventually resulting in HSCs differentiation into CAFs. In contrast, blockade of this CXCR4/TGF-β1 axis inhibits hepatic CAFs differentiation and CRC metastases to the liver (Tan et al., 2020). These findings seemingly underscore a critical role of TGF-β in mediating the generation of CAFs derived from not only fibroblasts but also non-fibroblasts in CRC. Interestingly, some histological observations have described that the source of CAFs in CRC can also be ascribed to MCs achieved via a mesothelial-to-mesenchymal transition (MMT) (Gordillo et al., 2020). Nevertheless, how MCs undergo MMT and following conversion into CAFs remains largely unclear in CRC. An RNA-sequencing analysis has revealed that the TGF-β signaling is related to MMT (Rynne-Vidal et al., 2017). It would be intriguing to test the possibilities that TGF-β may also be involved in MMT-mediated differentiation of MCs into CAFs in CRC.

It has been established that the MSCs have the potential to differentiate into mesenchymal tissues like osteocytes, chondrocytes, and adipocytes. They also have a differentiation potential beyond the mesenchymal lineage, such as myogenic, cardiomyogenic, and neurogenic potentials (Jackson et al., 2007). Besides, the MSCs were found to be differentiated into ECs (Oswald et al., 2004) and deeply associated with HSCs (Kordes et al., 2013). Further, the fibroblasts share many similarities between MSCs, including differentiation potential (Haniffa et al., 2009; Soundararajan and Kannan, 2018). Hence, the tight relationships between these cells may possibly influence the pool of cellular precursors of CAFs, whereby affecting the generation of CAFs in CRC. Nevertheless, it should be noted that given the original heterogeneity of CAFs, the sources of CAFs in CRC may not be limited to the above-described precursor cells (Table 1). Techniques like the lineage tracing, a powerful tool of deciphering cell-fate decisions (Kretzschmar and Watt, 2012), are expected to be employed in future studies to identify other cellular origins of CAFs in CRC, which would be very helpful to understand the complex nature of CAFs in CRC in the TME.

## Markers of Cancer-Associated Fibroblasts in Colorectal Cancer

A number of markers that are highly expressed in CAFs, such as the α-SMA, fibroblast activation protein alpha (FAP), fibroblast-specific protein 1 (FSP-1), platelet-derived growth factor receptor-α (PDGFRα) and PDGFRβ, have already been widely used to identify or isolate CAFs from the pool of fibroblasts present in the whole body (Nurmik et al., 2020). However, a critical issue remains as CAFs are composed of heterogeneous population of cells, and accordingly, markers of CAFs are vastly heterogeneous in different CAF subpopulations and consequently show low specificity. To date, there are no specific or reliable markers for CAFs in various tumors. Despite this dismay, many progresses have been witnessed over the last decade in seeking potential markers of CAFs in CRC and elucidating their relations to disease progression (Table 2). For example, the cell-surface molecule CD10 (Zhu et al., 2016) and the interleukin (IL)-11 (Nishina et al., 2021) might serve as possible markers of CAFs in CRC, although more lines of evidence are required to consolidate this possibility. Theoretically, candidate biomarkers of CAFs may be those molecules displaying significantly different expression levels between CAFs and normal counterparts. It is well accepted that compared with normal fibroblasts, differences in genetic, epigenetic, morphology and secretions are evident in CAFs in CRC (Mrazek et al., 2014; Wen et al., 2015). A proteome profiling of CAFs and normal fibroblasts purified from colon tissues has identified LTBP2, CDH11, OLFML3, and FSTL1 as selective biomarkers of CAFs (Torres et al., 2013). Aside from these proteins, CAFs from colon tissues of CRC patients show increased expression in several species of a disintegrin and metalloproteinases (ADAMs), including ADAM9, ADAM10, ADAM12, and ADAM17 (Mochizuki et al., 2020), as compared with normal fibroblasts. Moreover, normal fibroblasts and CAFs have significant differences in their protein expression profiles among 7 patient pairs, with 145 differentially expressed proteins revealed by the proteomic data, and 15 differentially expressed molecules shown by a secretomic analysis (Atanasova et al., 2020). Interestingly, by performing the next generation sequencing, a significant number of non-coding RNAs (ncRNAs) in exosomes were also found as potential biomarkers present in CAFs-derived exosomes (Herrera et al., 2018). Furthermore, a differential secretome approach of CAFs and bone marrow-derived precursors has identified in clinical CRC specimens a series of candidate biomarkers such as tenascin C, fibronectin ED-A domain and stromal-derived factor-1 (SDF1) that are associated with a CAF-specific phenotype (De Boeck et al., 2013). These comparative studies replenish the repository of candidate markers of CAFs in CRC, which need verifications by more investigations.

**TABLE 2:** Candidate markers of CAFs in CRC.

Name	Description	Confirmed material	References>
IL-11	IL-6 family cytokine	Animal CRC model	Nishina et al., 2021
CD10	Cell surface zinc metalloendopeptidase	Human CRC specimen	Cui et al., 2010; Zhu et al., 2016
LTBP2 CDH11 OLFML3 FSTL1	ECM protein Adhesion molecule ECM-related protein Extracellular glycoprotein	Animal CRC model; Human and mouse CRC specimen	Torres et al., 2013
ADAMs	Proteases	Human CRC specimen	Mochizuki et al., 2020
Exosomal ncRNAs	RNA molecules	Human CRC specimen	Herrera et al., 2018
Tenascin C ED-A FN SDF1	ECM glycoprotein ECM protein Chemokine	Human CRC specimen	De Boeck et al., 2013

*ECM, extracellular matrix; CDH11, cadherin-11; ADAMs, a disintegrin and metalloproteinases; ncRNAs, non-coding RNAs; ED-A FN, fibronectin ED-A domain; SDF1, stromal-derived factor-1.*

Along with the appearance of a growing body of potential markers of CAFs, accumulating evidence has also related some markers to the roles involved in CRC progression. Collagen I, PDGFR-β and α-SMA have been known as molecular markers of CAFs, and in advanced CRC, their expression varies in CAFs and is significantly associated with vessel markers CD31 and CD34, indicating that individual CAFs may have different expression patterns and effects on venous invasion of advanced CRC (Nishishita et al., 2018). In consistence, the expression of CAFs markers, like α-SMA, CD10, podoplanin and FSP1, is correlated with lymph node metastasis in the submucosal invasive CRC, therefore may allow for stratification of patients with high risk of lymph node metastasis (Sugai et al., 2018). Furthermore, a transcriptomic analysis has also shown that CAF markers, such as α-SMA, PDGFR-β, FAP, FSP-1, are expressed in a higher level in stroma-high compared to stroma-low CRC tissues, particularly with higher FAP expression in the invasive part of tumors (Sandberg et al., 2019), together suggesting that these molecular markers, as indicative of CAFs, could play a promotive role in CRC progression.

Although the research approaches mentioned above have yielded a cohort of promising candidate markers for CAFs, novel selection methods based on cellular functions such as lineage tracing and single-cell sequencing will be preferable for improving the identification and targeting of CAFs in CRC in a more specific manner.

## Cancer-Associated Fibroblasts in Colorectal Cancer Prognosis

Cancer-associated fibroblasts accumulated in large numbers in the TME are often associated with high-grade malignancies and poor prognosis across different human cancers. The prognostic impact of CAF-derived markers or gene signatures has also been demonstrated in CRC (Herrera et al., 2013b; Paulsson and Micke, 2014). For example, the expression of CAF markers, including α-SMA, FSP1, and FAP, is associated with the clinical outcome of a cohort of 289 CRC patients, and surprisingly, the combination of these CAF markers with M2 macrophage markers, CD163 and DCSIGN, identifies significant differences in the survival of advanced-stage patients, demonstrating a prognostic involvement of interrelationships between markers of CAFs and M2 macrophages in CRC patient survival (Herrera et al., 2013a). Specifically, the common and high intratumoral expression of FAP is associated with poorer prognosis of CRC patients, which emphasizes FAP as an independent negative prognostic factor (Wikberg et al., 2013). In general, CAFs serve as a useful prognostic biomarker in CRC, but it should be noticed that podoplanin, α-SMA or S100A4 expressing CAFs have been shown to be associated with different prognosis in CRC (Choi et al., 2013), which possibly indicate varying prognostic significances conferred by different populations of CAFs. On the other hand, a CAF-derived 5-gene classifier selected from 108 differentially expressed genes, including CCL11, PDLIM3, AMIGO2, SLC7A2, and ULBP2, is significantly associated with increased relapse risk and death from CRC across all validation series of stage II/III patients (Berdiel-Acer et al., 2014). In addition, a recent study has reported that the 1,25-dihydroxyvitamin D3 [1,25(OH)2D3]-associated gene signature in CAFs predicts a favorable clinical outcome in CRC (Ferrer-Mayorga et al., 2017). This association may be explained by a protective effect of the active vitamin D metabolite 1,25(OH)2D3 against CRC via regulation of CAFs. Besides, a CAF-related gene osteopontin (OPN) was also found to be a predictive biomarker for metastatic CRC patients treated with first-line FOLFIRI/bevacizumab in two independent randomized phase III trials (Puccini et al., 2018). Moreover, GREM1 and ISLR are newly identified CAF-specific genes, and their stromal high levels in CRC patients are associated with poor and favorable survival, respectively, which is mechanistically attributed to their inverse regulation of the bone morphogenetic protein (BMP) signaling in the stroma (Kobayashi et al., 2021). This finding also suggests that the status of this pathway could be considered as a predictive factor for CRC survival.

Apart from CAF markers or gene signatures, accumulating studies also have revealed other prognostic markers that are expressed in CAFs of CRC. In an immunohistochemical evaluation of 110 CRC patient cases, the ubiquitin carboxyl-terminal hydrolase L1 (UCH-L1) in CAFs was shown to be an independent prognostic factor for predicting shorter survival and a higher incidence of recurrence and lymph node metastasis (Akishima-Fukasawa et al., 2010). Additionally, protein expression of the lysyl oxidase-like 2 (LOXL2) in CAFs of CRC was identified to be associated with poor outcome of CRC patients and as a prognostic biomarker particularly for stage II patients (Torres et al., 2015). Further, the expression of an immune checkpoint molecule CD70 was detected on the majority of CAFs in invasive CRC specimens and shown significantly correlated with clinicopathological parameters such as metastasis, differentiation and advanced stage, and consequently, CD70-positive CAFs were defined as poor prognostic markers for CRC (Jacobs et al., 2017). In concert, another immunohistochemical evaluation of 269 primary CRCs also uncovers that CAFs exhibit various CD70 expression, which predicts worse survival in CRC patients (Inoue et al., 2019). CAFs are known to secrete different cytokines. One study using a cytokine chip has found that CAFs in CRC secrete the c-type lectin domain family 3 member B (CLEC3B), and that CRC patients with combined expression of CLEC3B and α-SMA have worse survival than those with either CLEC3B or α-SMA expression alone (Zhu et al., 2019), offering CLEC3B as a potential valuable CAF-based biomarker for CRC prognosis. Furthermore, some proteins deregulated in the CAFs of CRC also show significant prognostic value. In distant metastases, PTEN expression in CAFs was detected lost in some CRC patients, which was linked closely to a worse prognosis (Kwak et al., 2014). On the contrary, another report has documented that STAT3 is activated in CAFs of human CRC, and pSTAT3 expression in CAFs is negatively correlated with the survival of CRC patients, illustrating it as a prognostic marker (Heichler et al., 2020). Together, these numerous studies as outlined above reinforce the concept that CAFs and CAF-derived factors have a prognostic significance in human CRC (Table 3).

**TABLE 3:** Prognostic impact of CAFs in CRC patients.

Name	CRC prognosis	Clinical case	References
**CAF marker**			
α-SMA	Poorer DFS and OS	591	Choi et al., 2013; Herrera et al., 2013a
FSP1	Poorer DFS and OS	289	Herrera et al., 2013a
FAP	Poorer DFS and OS	738	Herrera et al., 2013a; Wikberg et al., 2013
Podoplanin, S100A4	Poorer DFS and OS	302	Choi et al., 2013
**CAF gene signature**			
CCL11, PDLIM3, AMIGO2, SLC7A2, ULBP2	Poorer DFS	108	Berdiel-Acer et al., 2014
Vitamin D receptor	Better PFS and OS	658	Ferrer-Mayorga et al., 2017
Osteopontin variant	Better DFS and OS	451	Puccini et al., 2018
GREM1	Poorer DFS and OS	556	Kobayashi et al., 2021
ISLR	Better DFS and OS	556	Kobayashi et al., 2021
**CAF-derived protein**			
UCH-L1	Poorer RFS and OS	110	Akishima-Fukasawa et al., 2010
LOXL2	Poorer DFS and OS	121	Torres et al., 2015
CD70	Poorer OS	269	Inoue et al., 2019
CLEC3B	Poorer OS	225	Zhu et al., 2019
PTEN	Better OS	181	Kwak et al., 2014
pSTAT3	Poorer OS	375	Heichler et al., 2020

*DFS, disease-free survival; OS, overall survival; PFS, Progression-free survival; RFS, recurrence-free survival.*

## The Versatile Roles of Cancer-Associated Fibroblasts in Colorectal Cancer

**FIGURE 1:**
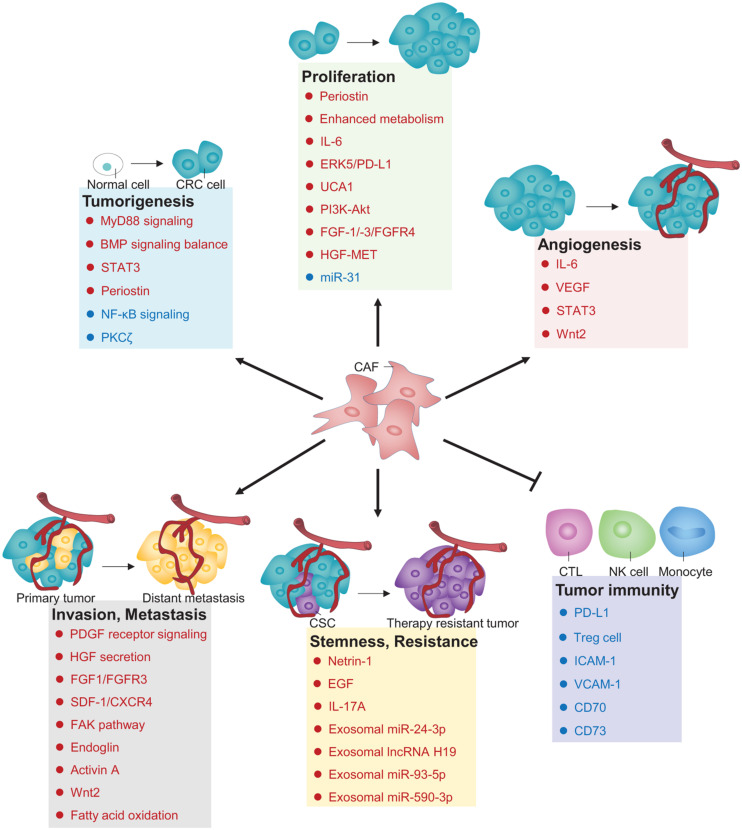
Principal roles and related mechanisms of CAFs in CRC hallmarks. Those depicted in deep blue are negative regulators, whereas those depicted in red are positive regulators controlling CAFs’ roles in CRC. CTL, Cytotoxic T lymphocyte; NK cell, natural killer cell; PD-L1, programmed death ligand 1; Treg cell, regulatory T cell; ICAM-1, Intercellular adhesion molecule-1; VCAM-1, Vascular cell adhesion molecule-1.

Cancer-associated fibroblasts are indispensable architects in the TME that play fundamental roles to radically influence multiple malignant behaviors. Over the recent decade, increasing lines of evidence have revealed the versatility of CAFs in CRC biology, including tumorigenesis, proliferation, angiogenesis, invasion and metastasis, stemness, therapy resistance, and tumor immunity (Figure 1). In this section, we will discuss these pivotal roles of CAFs in the regulation of pathogenic processes during CRC development and progression.

### Tumorigenesis

It is well recognized that CAFs play a critical role in modulating tumorigenesis. However, the role and mechanism of CAFs in CRC tumorigenesis are still poorly understood. Emerging studies have unveiled several CAF-based regulators and signaling pathways that could control CRC development. For instance, the myofibroblast MyD88-deficient mice were found resistant to AOM/DSS-induced intestinal tumorigenesis, and the STAT3/PPARγ pathway activated by the MyD88 signaling in myofibroblasts was demonstrated to contribute to this phenotype (Yuan et al., 2021). Further, periostin, a multifunctional extracellular matrix protein, is induced in fibroblasts by STAT3 activation, which ultimately facilitates CRC development in AOM/DSS and genetically modified mice (Ma et al., 2020). It has also been recently shown that the constitutive activation of STAT3 in the CAFs of CRC promotes tumorigenesis, and in contrast, STAT3 inactivation reduces the development of CRC in a mouse model established with AOM/DSS (Heichler et al., 2020). Hence, these findings may identify a crucial role of CAFs’ STAT3 signaling in facilitating CRC tumorigenesis. In addition, the selective loss of PKCζ in colonic fibroblasts induces a CAF phenotype in vitro and promotes intestinal tumorigenesis *in vivo*, which depends on the activation of SOX2 that drives the generation of a CAF population (Kasashima et al., 2021). Moreover, the bone morphogenetic proteins (BMPs) are key growth factors secreted by CAFs. A recent study has discovered that the stromal BMP signaling balanced by GREM1 and ISLR functions to drive CRC carcinogenesis (Kobayashi et al., 2021). These findings provide further supportive evidence depicting CAFs as a positive regulators in assisting CRC development.

While CAFs have been generally perceived to be driving forces for tumorigenesis, they also retard tumorigenesis via largely unknown mechanisms (Gieniec et al., 2019). CAFs have been reported to support tumorigenesis through mediating tumor-enhancing inflammation in an NF-κB-dependent manner, and a proinflammatory NF-κB gene signature in CAFs promotes tumorigenesis in models of pancreatic and skin cancers (Erez et al., 2010). Nevertheless, using an animal model of colitis-associated cancer (CAC) and sporadic colon tumors initiated by AOM, lines of direct genetic evidence have been obtained, which uncover an unexpected tumor-suppressive role of NF-κB signaling in CAFs that confers anti-tumorigenic effects and suppresses intestinal tumorigenesis *in vivo* (Pallangyo et al., 2015). Given the high plasticity in CAFs, this disparate finding may be attributed to distinct functions of NF-κB signaling depending on the activation status of CAF subpopulations. In any case, these results shed new light on the CAF regulation of CRC tumorigenesis.

### Proliferation and Angiogenesis

Except cellular autonomous properties, the progression of malignant tumors also relies on the active involvements of CAFs. In a non-contact co-culture system, the conditioned media (CM) from CAF cultures was found to enhance the proliferation of CRC cells stronger than those from normal fibroblasts (Nakagawa et al., 2004). CAFs indeed promote proliferation of CRC in vitro and tumor-bearing mouse models *in vivo* (Li et al., 2019a). The proliferative advantage endowed by CAFs could be at least partially explained by the CAF-secreted periostin (Kikuchi et al., 2008), CAF-enhanced metabolism of CRC cells (Zhou W. et al., 2017), and CAF-derived IL-6 (Xu et al., 2021). The mechanistic insights into CAF-promoted CRC proliferation are provided by other non-negligible clues, which show that the microRNA-31 (Yang et al., 2016), the long non-coding RNA UCA1 (Jahangiri et al., 2019), and some signaling pathways, including PI3K-Akt (Yamamura et al., 2015), FGF-1/-3/FGFR4 (Bai et al., 2015), HGF-MET (Wen et al., 2020), and ERK5/PD-L1 signaling axes (Zhang M. et al., 2020), also act as important modifiers mediating the pro-proliferative effects of CAFs on CRC. These distinct molecular mechanisms support the notion that CAFs can form a favorable microenvironment for the proliferation of CRC cells. Instead, CRC cell-derived hydrogen sulfide was found to enhance CAF cell proliferation (Coletta et al., 2014), possibly postulating a reciprocal interaction between CAFs and CRC cells that may enhance the tumor cell proliferation more robust.

Tumor angiogenesis establishes new microvessels that support cancer cell proliferation by providing nutrients and oxygen. During this complex process, many angiogenic factors, especially the vascular endothelial growth factor (VEGF), play a vital role (Lugano et al., 2020). In CRC tissues, CAFs are important sources of IL-6, which enhances VEGF production, whereby inducing tumor angiogenesis (Nagasaki et al., 2014). In accordance with this, the eicosapentaenoic acid was proved to suppress CRC angiogenesis via reducing the secretion of IL-6 and VEGF from CAFs (Ando et al., 2019). Moreover, it has been shown that IL-6-activated STAT3 in fibroblast subpopulations regulates the transcriptional patterns associated with angiogenesis, and blockade of proangiogenic signaling impedes CRC growth in genetically modified mice with constitutive STAT3 activation in fibroblasts (Heichler et al., 2020). This study suggests that STAT3 might be a downstream target that mediates the proangiogenic effect of CAF-produced IL-6 on CRC. Except IL-6, CAFs-derived Wnt2 can also increase tumor angiogenesis in CRC, owing largely to Wnt2-upreglated expression of some proangiogenic proteins (Unterleuthner et al., 2020). Based on these discoveries, it is tempting to speculate that the transcriptional reprogramming initiated by CAF-secreted IL-6 or Wnt2 could shift the balance toward proangiogenic signals in favor of tumor angiogenesis and proliferation.

### Epithelial-to-Mesenchymal Transition, Migration, Invasion, and Metastasis

The malignant progression of cancer is a dynamic process depending not solely on genetic alterations, but also on additional regulations by the TME (Brabletz et al., 2005). A molecular profiling analysis of CAFs isolated from human CRC has delineated them as major participators in promoting CRC metastasis (Potdar and Chaudhary, 2017). The maturity of CAFs was also associated significantly with cancer invasion for CRC patients (Shin et al., 2019). Moreover, an earlier study has reported that compared with the CM of normal colonic fibroblasts or CAFs from primary tumors, the CM of CAFs from liver metastasis leads to more aggressive phenotypes, including the epithelial-to-mesenchymal transition (EMT), migration and invasion (Berdiel-Acer et al., 2011). These reports suggest that CAFs serve to accelerate the malignant progression of CRC. Yet, the functional contributions to this process and the molecular mechanisms are not fully clear.

In recent years, increasing studies has indicated that an intense biochemical cross-talk between CRC cells and CAFs is forged by the CAF-secreted numerous factors, which is critical for tumor progression into a metastatic malignancy. For example, the secreted glycoprotein stanniocalcin-1 (STC1) was identified to mediate the function of the platelet-derived growth factor (PDGF) receptor signaling in increasing the migration, invasion and metastasis of CRC (Pena et al., 2013). Typically, the hepatocyte growth factor (HGF) can activate cancer cell invasion and metastasis. Consistently, it was reported that the migration of CRC cells could be promoted by the Ras-related protein Rab-31 (RAB31) through regulating HGF secretion in the tumor stroma (Yang et al., 2020). Human CRC-derived CAFs also enhance the adhesion of CRC cells to ECs by secretion of HGF (Zhang et al., 2019a). Additionally, HGF contributes to EMT induction in CRC cells by CAFs’ secretomes (Wanandi et al., 2021). Moreover, studies have shown that CAFs secrete the fibroblast growth factor 1 (FGF1) to increase CRC cell invasion via FGFR3 signaling (Henriksson et al., 2011), as well as the stromal cell-derived factor-1 (SDF-1) to promote CRC metastasis to distant organs via the C-X-C chemokine receptor type 4 (CXCR4) axis (Peng et al., 2018). Combining another research which shows that by secreting the LOXL2, CAFs stimulate the focal adhesion kinase (FAK) pathway and consequently induce the EMT and metastasis of CRC cells (Xuefeng et al., 2020), those research progresses characterize these signaling as vital mediators in transducing CAFs’ notorious effects on malignant behaviors of CRC. Other CAF-secreted factors that have recently been shown to promote the EMT, migration and invasion of CRC include the CLEC3B (Zhu et al., 2019), activin A (Bauer et al., 2020), and Wnt2 (Aizawa et al., 2019). However, how Wnt signaling regulates CRC progression is still in controversies, since a phenotypic switch of CAFs induced by Wnt was reported to inhibit EMT in CRC, implying that the Wnt signaling may induce subtypes of CAFs with differential activities in CRC progression (Mosa et al., 2020). Furthermore, CAFs-derived exosomal miR-17-5p (Zhang Y. et al., 2020) and LINC00659 (Zhou et al., 2021) were found to promote CRC metastasis, and invasion and migration, respectively. These advancements also manifest that CAFs could promote CRC progression through secreting exosomes to influence adjacent cancer cells.

Recently, growing study efforts have been devoted to understanding how metabolic reprogramming is mechanistically involved in CAF-promoted CRC progression. In orthotopic CRC models, the activated CAFs have been discovered to promote a metabolic switch favoring glutamine consumption in CRC cells, which results in increased number of organ metastases (Tommelein et al., 2018). On the other side, CAFs were uncovered to undergo a lipidomic reprogramming in order to accumulate and accordingly secrete more fatty acids, and CRC cells were confirmed to take up these lipids metabolites, eventually leading to their potentiated migration (Gong et al., 2020). Keeping in line with this, CAFs were proved to promote the migration and invasion of CRC cells, and drive the peritoneal metastasis via activating fatty acid oxidation and modulating glycolysis (Peng et al., 2021). These results provide further insights into CRC progression regulated by CAFs.

Some divergent mechanisms also emerge to underlie the roles of CAFs in CRC progression. At the invasive borders of CRC, CAFs specifically express endoglin, with its levels correlated positively with disease stages and poor metastasis-free survival. Functionally, endoglin neutralization inhibits CRC cell invasion *in vitro* and decreases metastatic spread of CRC cells to the liver (Paauwe et al., 2018), suggesting a significant role of endoglin-expressing CAFs in promoting CRC progression. Moreover, the activation of RNA editing of the antizyme inhibitor 1 (AZIN1) in CAFs was revealed to enhance the invasive potential of CAFs in CRC (Takeda et al., 2019). It is also worth to note that CAFs can induce CRC cell migration and invasion by a contact-dependent manner (Knuchel et al., 2015). Actually, CRC cells are not bystanders upon encountering the interaction with CAFs. It has been shown that CRC cells express the structural maintenance of chromosomes 1A (SMC1A), a subunit of cohesion, which functions to recruit CAFs, whereby promoting CRC metastasis (Zhou P. et al., 2017). Additionally, when interacting with CAFs, the activation of the phospholipase D in CRC cells is required to mediate the pro-migration effects (Majdop et al., 2018). However, it remains unclarified how SMC1A and phospholipase D exert their functions during these processes. Another clue is that in the circulation system, the tumor cell clusters play a primary role in cancer metastasis, which can be enhanced by the interaction with clusters of CAFs (Hurtado et al., 2020). It would be therefore very tempting to test whether SMC1A and phospholipase D regulate cluster interactions between CRC cells and CAFs, whereby enhancing the effect of CAFs on CRC metastasis.

### Stemness and Therapy Resistance

Cancer-associated fibroblasts stimulate an EMT-driven gain of cancer stemness through a paracrine interplay between CAFs and prostate cancer cells (Giannoni et al., 2010), and also constitute a supporting niche for cancer stemness in lung cancer through a paracrine IGF-II/IGF1R signaling (Chen et al., 2014). These studies indicate that CAFs can maintain cancer stemness in some cancer types. In truth, this function of CAFs can be applied to CRC as well, wherein CAFs can upregulate netrin-1 to increase its stemness in vitro and in mice (Sung et al., 2019). Being clinically relevant, the expression of CRC stemness markers are also upregulated by CAF secretomes from CRC patients (Wanandi et al., 2020). It has also been documented that CAFs promote CRC stemness by transferring exosomal lncRNA H19, which acts as a miR-141 sponge to suppress its inhibitory effect on stemness (Ren et al., 2018), therefore consolidating the role of CAFs in increasing stemness in CRC. Similarly, this mechanism is analog to that found in a variety of solid tumors, with their stemness regulated by CAF-secreted exosomes (Huang et al., 2019).

Functionally, cancer stem cells (CSCs) are believed to be a driving force behind tumorigenesis and also play major roles in tumor resistance and recurrence. In CRC patients, a significant increase in the number of CAFs was observed after cytotoxic treatment, and CSCs were shown to be promoted by CAFs via augmented secretion of specific cytokines, including IL-17A, which in turn lead to increased resistance to chemotherapy (Lotti et al., 2013). Coincidently, it is established that CAFs could promote chemoresistance by supporting a niche to sustain cancer stemness (Su et al., 2018). In regard to CRC resistance to chemotherapeutics, CAFs and CAF-derived exosomal miR-24-3p have been validated to accelerate resistance of CRC cells to oxaliplatin, 5-fluorouracil and methotrexate (Goncalves-Ribeiro et al., 2016; Yadav et al., 2020; Zhang et al., 2021). Also, in the presence of CAFs, tumor cells show reduced sensitivity to cetuximab, a monoclonal antibody therapy targeting the epidermal growth factor receptor (EGFR) (Garvey et al., 2017). A recent finding has discovered that cetuximab increases CAFs’ EGF secretion, which is sufficient to render neighboring cancer cells resistant to cetuximab in combination with chemotherapy for metastatic CRC patients (Garvey et al., 2020). Moreover, CAF-derived exosomal miR-93-5p or miR-590-3p has been shown to rescue CRC cells from radiation-induced apoptosis (Chen et al., 2020, 2021), and the promoted CRC stemness was demonstrated to account for the radioresistance imposed by CAF-derived exosomes (Liu L. et al., 2020). Thus, these discoveries build a mechanistic connection between CAF-maintained cancer stemness and therapy resistance in CRC.

### Tumor Immunity

The immunomodulatory effects of CAFs on CRC have been observed in a progressive rat model, in which T lymphocytes and monocytes were found outside the myofibroblast-surrounded tumors (Lieubeau et al., 1999). Recent findings have also shown that the levels of CAFs markers such as α-SMA, thrombin and fibronectin are significantly higher in CRC than in normal colonic mucosa, and α-SMA expression is negatively correlated with the number of tumor-infiltrating lymphocytes (TILs), while fibronectin displays positive coexpression (Zadka et al., 2021), and that CAF phenotypes are also correlated with CD8^+^ T-cell infiltration (Johnson et al., 2020), hence underscoring the importance of CAFs in regulating CRC immunity. CAFs are also positively correlated with PD-L1 expression in CRC tissues, and through secreting CXCL5, CAFs are able to promote PD-L1 expression in cancer cells (Li et al., 2019b). And moreover, a significant association has been validated between elevated Treg amounts and CD70-expressing CAFs (Jacobs et al., 2017). These observations illustrate CAFs as regulators of tumoral immunosuppression of the T cell response.

Monocytes affect the TME and induce immune tolerance (Ugel et al., 2021). CAFs have been shown to increase the recruitment of monocytes into the CRC TME via various mechanisms. Firstly, CRC CAFs exhibit upregulated ICAM-1 expression and affinity for monocytes, as such, increasing their interaction to elongate monocyte residence in CRC tissues (Schellerer et al., 2014). Secondly, CRC CAFs promote the adhesion of monocytes by upregulating VCAM-1 expression in CRC cells. Thirdly, CAFs can also attract monocytes by secreting IL-8 (Zhang et al., 2019b). Subsequently, CAFs promote M2 polarization of macrophages to suppress the activity of natural killer (NK) cells in CRC (Zhang et al., 2019b), favoring the escape from attack by the tumor immunity.

Notably, it has been reported that CAFs can regulate immune checkpoint in CRC. CAFs in human CRC tissues constitute the major population expressing CD73, a molecule acting as an immune checkpoint to suppress immune activation through the A2A receptor, and importantly, CD73 expression on CAFs is enhanced via A2B-mediated feedforward circuit triggered by tumor cell death, which enforces the CD73 immune checkpoint and consequently counteracts the antitumor immunity in CAF-rich CRC (Yu et al., 2020). Taken together, these immunosuppressive activities of CAFs on CRC have significant clinical impacts, rendering CAFs to be potential therapeutic biomarkers as well as targets for CRC.

## Cancer-Associated Fibroblasts as Therapeutic Targets in Colorectal Cancer Treatment

**FIGURE 2:**
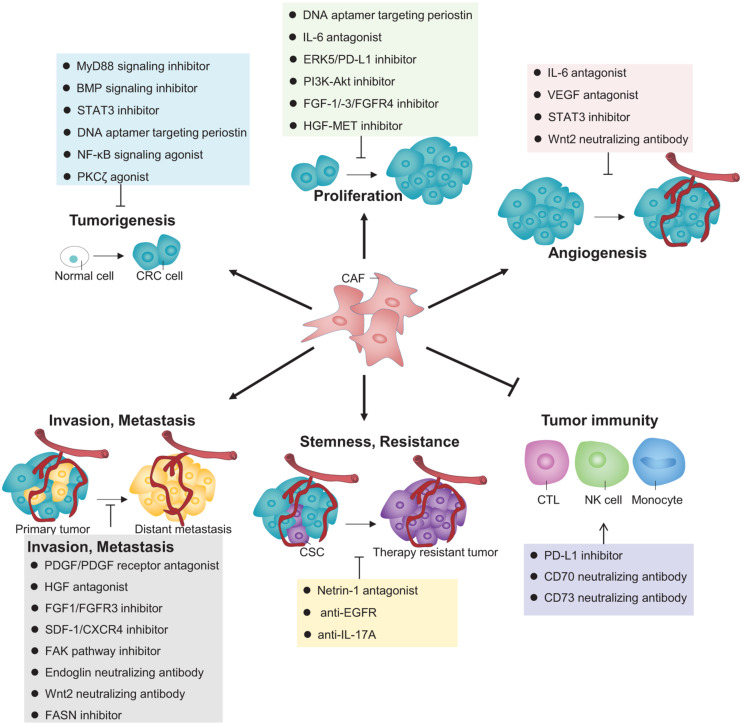
Therapeutic strategies that target CAFs for CRC treatment. A variety of inhibitors, agonists, or neutralizing antibodies targeting different signaling pathways or molecules that contribute to CAFs’ versatile roles are proposed to interfere some key processes during CRC pathogenesis for clinical treatment.

As discussed above, the increasingly deep understanding into the CAFs’ exquisite regulation of CRC pathogenesis achieved over recent years by pioneering studies has sparked vast inspirations to develop some potential mechanism-based targeted therapies, which can be classified according to their respective effects directed to each functional role of CAFs in impacting CRC, as illustrated in Figure 2.

A number of preclinical studies have described the tight control of CRC tumorigenesis by CAFs, implicating that there are some druggable targets in CAFs that possess the potentialities for CRC prevention or intervention. For example, MyD88 signaling in CAFs contributes crucially to colitis-associated CRC carcinogenesis via promoting macrophage M2 polarization (Yuan et al., 2021). Interestingly, interfering with this pathway by a novel synthetic inhibitor TJ-M2010-5 has been demonstrated to prevent colitis-associated CRC in mice (Xie et al., 2016). These clues suggest that inhibiting MyD88 signaling in CAFs with synthetic inhibitors may be used as a therapeutic modality for treating CRC. Moreover, CAF-secreted periostin is revealed to promote CRC tumorigenesis and proliferation (Kikuchi et al., 2008; Ma et al., 2020). Some clinical trials by inhibition of periostin function are ongoing to test its therapeutic effects on periostin-related diseases (Kudo, 2019). Encouragingly, an earlier study has shown that the benzyl-d(U)TP-modified DNA aptamers targeting human periostin inhibit breast cancer growth (Lee et al., 2013). Hence, these studies prompt that targeting periostin may inhibit CRC development. Moreover, through loss-of-function approaches, the constitutive activation of STAT3 in CAFs is also shown to accelerate CRC tumorigenesis in mice (Heichler et al., 2020). A series of STAT3 inhibitors and analogs have been identified and show considerable anti-CRC effects (Chalikonda et al., 2021). Hopefully, these agents could be exploited to suppress CRC tumorigenesis by inhibiting STAT3 in CAFs. Further, the enhanced CRC tumorigenesis *in vivo* by the deletion of CAFs’ PKCζ supports a revised paradigm holding a view that the PKC family acts as a tumor suppressor (Newton and Brognard, 2017). As a result, restoring rather than inhibiting PKCζ activity in CAFs could be a strategy to restrict CRC. The disruption of stromal BMP signaling using small molecule agonists/activators, such as DMH1, a highly selective small-molecule inhibitor of BMP receptor (Owens et al., 2015), also represents a possible avenue to interfere CRC development, since its balance could drive CRC carcinogenesis (Kobayashi et al., 2021).

Cancer-associated fibroblasts-secreted IL-6 and ensuing STAT3 activation promote CRC proliferation and angiogenesis. Because targeting IL-6 are effective in some inflammatory diseases in clinical trials (Kang et al., 2019). It is very tempting to assess whether therapeutic agents blocking IL-6 also yield satisfactory outcomes for CRC patients. In addition, inhibitors of signaling axes, including PI3K-Akt, FGF-1/-3/FGFR4, HGF-MET, and ERK5/PD-L1, also hold promise to combat CRC, due to the fact that they can mediate the pro-proliferative effect of CAFs on CRC. In the process of tumor angiogenesis in CRC tissues, CAFs-derived Wnt2 and its elevated proangiogenic signals play an important role. Besides, autocrine Wnt2 signaling in CAFs also promotes CRC progression (Kramer et al., 2017). Noticeably, targeting CAF-secreted Wnt2 was recently reported to restore anti-tumor immunity (Kang et al., 2019). These findings classify Wnt2 as a promising stromal target to confine CRC progression. Future studies are needed to test the effect of Wnt2 neutralizing antibodies toward CRC. The result may be predictably satisfactory, because an earlier study has already shown a therapeutic effect of an anti-Wnt2 monoclonal antibody against malignant melanoma (You et al., 2004).

The PDGF receptor signaling functions to transduce the pro-metastatic signals from CAFs into CRC cells, and inhibition of this signaling has proven useful for treating patients with some tumors (Heldin, 2013). Whether PDGF/PDGF receptor antagonists will be beneficial for reducing metastasis and prolonging survival for CRC patients is an ongoing and future study direction for the management of patients with metastatic CRC (Advani and Kopetz, 2019). Moreover, signaling pathways induced by HGF, FGF1, SDF-1, and FAK are profoundly involved in CAFs’ roles in enhancing the malignant behaviors of CRC, providing them as potential targets to obstruct disease progression. These topics have been intensively reviewed or discussed elsewhere (Tommelein et al., 2015; Jeong, 2018; Parizadeh et al., 2019). Additionally, the metabolic reprogramming in CAFs that aids to expedite CRC progression also offers attractive targets for therapeutic intervention. For instance, the fatty acids synthase (FASN) is crucial for fatty acids synthesis and is significantly increased in CAFs, which is responsible for CAF-induced CRC cell migration *in vitro* and *in vivo* (Gong et al., 2020). These results suggest that the FASN of CAFs may be a target for anti-metastasis in CRC treatment. Recently, the first FASN inhibitor (TVB-2640) has completed the phase 1 clinical trial for solid tumors (Angeles and Hudkins, 2016). This successful translation from bench to clinic may open new opportunities for expanding the utility of FASN inhibitors to inhibit CRC metastasis. Further, the selectively high expression of endoglin in CAFs and its correlation with metastasis and poor survival in CRC patients will lead to the exploration of testing it as a therapeutic target. The coincidence is that the endoglin neutralizing antibodies such as TRC105 are being tested in clinical studies in cancer patients as a monotherapy or incorporated into combinatory therapies (Liu Y. et al., 2020). These studies will enable us to learn the prospect of treating malignant CRC through targeting endoglin-expressing CAFs.

The CAF-increased chemoresistance in CRC can be achieved via CSC self-renewal that is promoted by CAF-derived IL-17A, and accordingly, targeting IL-17A signaling impairs CSC growth and overrides chemoresistance (Lotti et al., 2013). The anti-IL-17A was approved by FDA for the treatment of psoriasis in 2015, proving its effectiveness clinically (Chen and Kolls, 2017). It therefore would be of clinical significance to examine the impact of anti-IL-17A on improving the efficacy of chemotherapy for CRC. The exploitation of immunomodulatory activities of CAFs as promising targets for CRC treatment cannot be ignored either. For example, by applying the therapeutic anti-EGFR humanized antibody cetuximab, the inhibition of NK cell function by the CAFs of CRC can be relieved (Costa et al., 2018), and neutralizing CD73 enhances antitumor immunity in CAF-rich CRC (Yu et al., 2020), therefore visualizing the therapeutic potential of the strategic targeting of CAF-mediated immune suppression.

Although the versatile pro-tumoral functions of CAFs performed during CRC development and progression make them to be attractive and promising therapeutic targets that can be harnessed for CRC treatment, the total depletion of CAFs unexpectedly results in more aggressive tumors (Ozdemir et al., 2014; McAndrews et al., 2021), demonstrating that different CAF subpopulations have opposite roles in cancer. Truly, CAFs in CRC tissues exhibit divergent phenotypes which can be differentiated at least by expression profiles and functions, as evidenced by transcriptional heterogeneity (Li et al., 2017) and functional heterogeneity (Herrera et al., 2013b). Since attempts to therapeutically target CAFs have been obstructed by our poor understanding of their heterogeneity (Kobayashi et al., 2021), future breakthroughs in translating basic sciences into CAF-based therapies will be witnessed with the better understanding of CAF heterogeneity, which can improve the therapeutic outcomes of cancer patients by targeting specific CAF subsets that promote cancer progression.

## Conclusion

Cancer-associated fibroblasts are crucial components of the TME which interacts intensively with proliferating tumor cells, together creating a developing tumor, including CRC. Currently, the first-line treatment options for advanced CRC are chemotherapy combined with targeted therapy. Despite some achievements in improving patients’ survival rates, the success of treatment is limited by targeting tumor cells alone. This dilemma has redirected more research attentions into investigating on the roles of the TME in the progression of CRC and their underlying mechanisms, in an effort to discover novel and more effective therapeutic strategies and targets for improving the available therapies. With the CAFs becoming the study focus, many advancements in our understanding of the CAF biology in CRC pathogenesis have been obtained in recent decade. We now know that the CAFs in CRC have heterogeneous precursors and markers, and also show a clinical significance in predicting patients’ prognosis. Mounting analyses in preclinical models have unveiled versatile roles and distinct mechanisms of CAFs that profoundly promote many key malignant behaviors of CRC, including tumorigenesis, proliferation, angiogenesis, invasion and metastasis, stemness and therapy resistance, and simultaneously attenuate tumor immune responses. These findings indisputably support the notion that CAFs can be considered as a prominent therapeutic target of stroma-based therapy in CRC treatment. However, targeting specific CAF subpopulation that promote cancer progression encounters a huge challenge in clinic, as little is known about a myriad of functions of different CAF subsets originated from their high heterogenetic nature. To address this difficulty, novel techniques like the lineage tracing and single-cell sequencing should be applied in the future to distinguish targetable subpopulations from the whole pool of CAFs within tumors. As such, the selective eradication of the tumor-promoting CAF subsets will be realized and then implemented in combination with the current therapeutic rationales for the better treatment of CRC and even other cancers.

## Author Contributions

LD and NJ conceived and wrote the manuscript. JZ helped with the table drawing. YW and HC reviewed and revised the manuscript. All the authors have read and agreed to the published version of the manuscript.

## Conflict of Interest

The authors declare that the research was conducted in the absence of any commercial or financial relationships that could be construed as a potential conflict of interest.

## Publisher’s Note

All claims expressed in this article are solely those of the authors and do not necessarily represent those of their affiliated organizations, or those of the publisher, the editors and the reviewers. Any product that may be evaluated in this article, or claim that may be made by its manufacturer, is not guaranteed or endorsed by the publisher.
